# A Toolkit for High Resolution Imaging of Cell Division and Phytohormone Signaling in Legume Roots and Root Nodules

**DOI:** 10.3389/fpls.2019.01000

**Published:** 2019-08-02

**Authors:** Marcin Nadzieja, Jens Stougaard, Dugald Reid

**Affiliations:** Department of Molecular Biology and Genetics, Aarhus University, Aarhus, Denmark

**Keywords:** auxin, cytokinin, nodulation and N fixation 2, lotus japonicus, symbiosis

## Abstract

Legume plants benefit from a nitrogen-fixing symbiosis in association with rhizobia hosted in specialized root nodules. Formation of root nodules is initiated by *de novo* organogenesis and coordinated infection of these developing lateral root organs by rhizobia. Both bacterial infection and nodule organogenesis involve cell cycle activation and regulation by auxin and cytokinin is tightly integrated in the process. To characterize the hormone dynamics and cell division patterns with cellular resolution during nodulation, sensitive and specific sensors suited for imaging of multicellular tissues are required. Here we report a modular toolkit, optimized in the model legume *Lotus japonicus*, for use in legume roots and root nodules. This toolkit includes synthetic transcriptional reporters for auxin and cytokinin, auxin accumulation sensors and cell cycle progression markers optimized for fluorescent and bright field microscopy. The developed vectors allow for efficient one-step assembly of multiple units using the GoldenGate cloning system. Applied together with a fluorescence-compatible clearing approach, these reporters improve imaging depth and facilitate fluorescence examination in legume roots. We additionally evaluate the utility of the dynamic gravitropic root response in altering the timing and location of auxin accumulation and nodule emergence. We show that alteration of auxin distribution in roots allows for preferential nodule emergence at the outer side of the bend corresponding to a region of high auxin signaling capacity. The presented tools and procedures open new possibilities for comparative mutant studies and for developing a more comprehensive understanding of legume-rhizobia interactions.

## Introduction

Plant hormones control all aspects of plant development. In legume plants, which enter into symbiotic relationship with soil bacteria called rhizobia, *de novo* formation of lateral organs called nodules must be coordinated with rhizobial infection for successful development of nitrogen fixing root nodules. These two separate, yet interdependent processes are regulated by plant hormones. Organ initiation and formation is restricted to a specific root zone and thus the spatiotemporal regulation of plant hormones is critical.

Studying plant hormone dynamics in this context requires high resolution sensors in order to discriminate signaling that regulate nodule organogenesis and infection processes. Several challenges have prevented the widespread use of hormone sensors in legume nodulation studies. Differences in promoter activity can for example impair the use of sensors developed in other model systems. Signal to noise ratios is also an issue and therefore nuclear localized variants of yellow and red fluorescent proteins are commonly employed in promoter studies instead of GFP (Suzaki et al., 2012; Reid et al., 2016). The “thick” multilayered anatomy of legume roots also pose challenges for deep imaging. However, recent advent of fluorescent compatible clearing methods which rely on fixation of fluorescent proteins prior to chemical clearing of tissue has facilitated the application of fluorescent markers in intact tissue (Warner et al., 2014).

The major plant hormones auxin and cytokinin have important roles in both nodule organogenesis and infection (Tirichine et al., 2006, 2007; Murray et al., 2007; Suzaki et al., 2012; Breakspear et al., 2014; Reid et al., 2017; Liu et al., 2018; Nadzieja et al., 2018) and we therefore focused our tool-box development on these two hormones. Transcriptional (promoter based) markers have traditionally been used, however present problems associated with gene silencing (Zürcher et al., 2013). Where available, translational markers including sensors based on ubiquitin-dependent degradation (DII, CycB) can significantly improve resolution and sensitivity (Colón-Carmona et al., 1999; Brunoud et al., 2012). Auxin and cytokinin are major regulators of the cell cycle that is activated during nodule organogenesis and we therefore aimed to characterize reporters for assessing cell cycle progression.

Here, we present a suite of tools for the study of auxin, cytokinin and cell cycle progression in legumes and demonstrate their use in the model legume *L. japonicus*. The toolbox consists of GoldenGate compatible parts (Patron et al., 2015) for rapid cloning and application. Additionally, we show that the fluorescent compatible clearing methodology provides a great improvement in imaging depth in multicellular tissues and enables comprehensive imaging of anatomical features and cell division during root and nodule development.

## Results

### Fluorescent Compatible Clearing and Cell Type Identification

**Figure 1:**
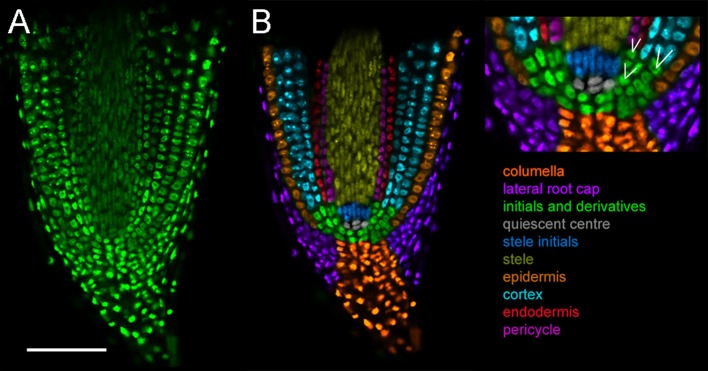
Cell types in *L. japonicus* root tip. Hairy roots transformed with *Lj*Ubi:tYFPnls and cleared for 10 days. Whole mount roots. **(A)** Original confocal section of median longitudinal section of the root tip **(B)** recoloured showing cell types. White lines indicate cell division sites generating new cell layers. Scale bar 100 μm.

Visualization of cortical cells in intact tissue is important for nodule developmental studies. Compared to *Arabidopsis thaliana, L. japonicus* roots display a multilayered, complex architecture. This impairs detection of fluorescence, making it difficult to image deeper cell layers at high resolution without physical sectioning. To assess the utility of the fluorescent compatible clearing method previously developed for pea nodules (Warner et al., 2014), we expressed nuclear localized triple venus YFP (tYFPnls) under a strong constitutive promoter (*Lj*Ubiquitin). Harvested roots were fixed using 4% *p*-formaldehyde followed by 10-day clearing in 6M urea. The fluorescent compatible clearing procedure offered a great improvement in imaging depth relative to uncleared roots (Figure S1). Cleared roots allowed identification of all cell files within the closed-type root apex of *L. japonicus* (Potocka et al., 2011) including (i) columella, (ii) lateral root cap, (iii) stele, (iv) epidermis, (v) cortex, (vi) endodermis, and (vii) pericycle (Figure 1). Cell types of different identity were recolored for ease of visualization (Figure 1B). This imaging also enabled identification of the quiescent center (QC) and initials forming pericycle, endodermis and cortical cells and cells giving rise to additional cortical cell layers (Figure 1B, box).

Fluorescent compatible clearing also improves imaging depth outside the area of the root apical meristem, but the application may be limited for lowly expressed fluorescent proteins. We cleared LjDII (see below) transgenic root (Supplemental Video 1) and were able to identify nuclei in all cortical layers. Intensity of the fluorescent signal deeper in the tissue is however greatly reduced. In young, infected nodules (6 dpi, Supplemental Video 2) it is possible to visualize large networks of infection threads with clarity enabling the pinpointing of infection thread branching points within a nodule.

### Application of Cytokinin Response Reporter TCSn in Nodule Development

Cytokinin signal transduction results in activation of transcription via phosphorylation of B-type response regulators (RRs). DNA binding domains of B-type Arabidopsis RRs (ARRs) are highly conserved and bind a common DNA motif (A/G)GAT(T/C) (Sakai et al., 2001; Hosoda et al., 2002; Imamura et al., 2003). This motif was used to create the synthetic cytokinin response reporter called TCS (Two Component Signaling) (Müller and Sheen, 2008), while optimization of the number, sequence and spacing between ARR-binding motifs was termed TCSn (TCS *new*; Zürcher et al., 2013). TCSn shows reduced sequence conservation to reflect a broader spectrum of naturally occurring ARR binding sites, resulting in higher sensitivity of the promoter. Reduced sequence repetition also lowers silencing of the reporter.

**Figure 2:**
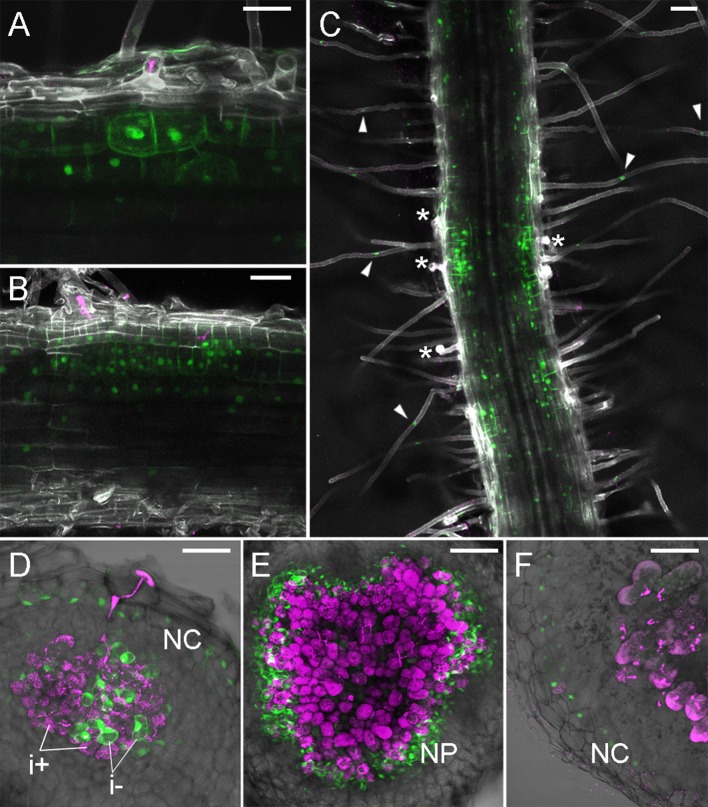
TCSn:YFPnls activation in nodule development. Plants were inoculated with *M. loti* MAFF303099 DsRed (magenta). **(A,B)** 4 dpi nodule primordia, **(C)** roots at 5 dpi **(D)** 7 dpi nodule, **(E)** 10 dpi nodule, **(F)** 14 dpi nodule. **(A–C)** Whole mounts, cleared roots, **(D–F)** 80 μm vibratome sections. Arrowheads—epidermal TCSn activation, asterisks—epidermal infection threads. NC, nodule cortex; NP, nodule parenchyma; i+/i-, infected/uninfected nodule cells. Scale bars 50 μm.

We previously described TCSn activity upon rhizobial inoculation over initial steps of symbiosis (Reid et al., 2017), showing a broad early activation of cytokinin signaling in the root cortex of the plant. Here, in subsequent stages of nodule development, we observed strong TCSn:YFPnls activation during establishment of nodule primordium and its subsequent proliferation, with its maxima in dividing cortical cells (Figures 2A,B). Nuclei directly below the infection thread appear enlarged, possibly due to endoreduplication (Suzaki et al., 2014) We also observed that epidermal, root hair activation of cytokinin signaling occurs in cells which do not contain infection threads (Figure 2C). In nodules at the stage of bacteria release into symbiosomes, TCSn is not active in infected cells, but remains active in interstitial uninfected cells (Figure 2D). Similarly, in fully developed nodules TCSn activity is not detectable in cells containing rhizobia, but in the outer nodule parenchyma (Figure 2E) and nodule cortex (Figure 2F). We did not observe TCSn activation in infected root hairs.

### Auxin Accumulation Reporters

Non-transcriptional auxin reporters have been developed based on breakdown of the auxin - Aux/IAA complex (Brunoud et al., 2012). This reporter, termed DII, includes the Aux/IAA degron from IAA28 cloned in frame with fast maturing yellow fluorescent protein (VENUS). Auxin mediated degradation of the DII-VENUS sensor results in a rapid decline of fluorescence. A mutated DII domain abolishing auxin binding (mDII) (Tan et al., 2007) allows for ratiometric imaging of auxin accumulation (R2D2; Liao et al., 2015).

**Figure 3:**
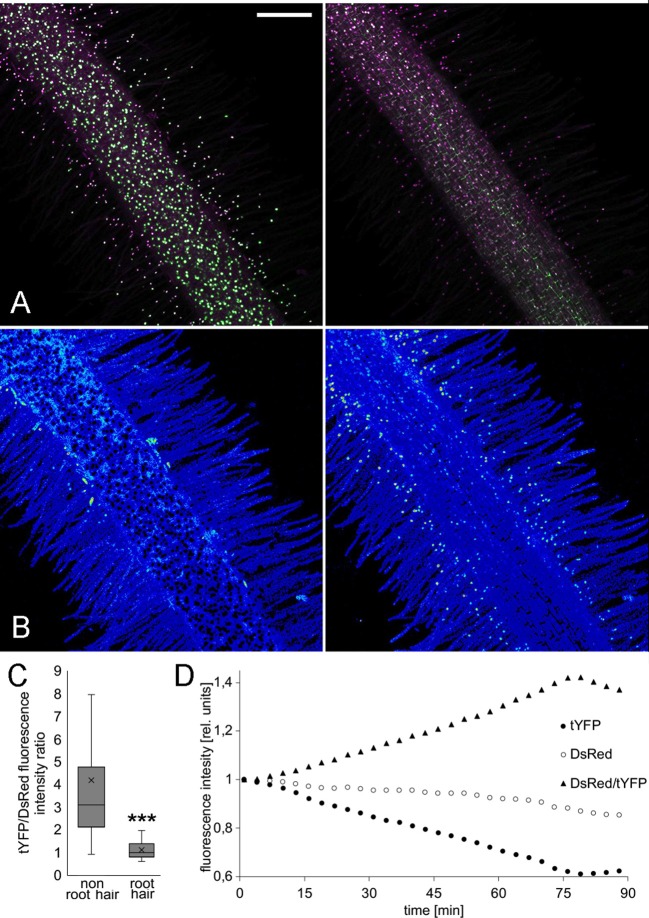
LjR2D2 response to exogenous IAA application. **(A)** tYFP (DII) and DsRed (mDII) fluorescence, original channels, **(B)** DsRed/tYFP fluorescence ratio, 18-colors LUT. **(A,B)** Left *t* = 1 min, **(A,B)** right *t* = 89 min. Scale bar 100 μm. **(C)** tYFP/DsRed fluorescence ratio in 30 root hair and 30 non root hair epidermal cells at the beginning of the treatment, **(D)** Graph representing change of tYFP and DsRed fluorescence intensities upon 10^−6^M IAA treatment. Whole mount live images are shown. ^***^p < 0.001.

We previously described a sensitive auxin accumulation reporter, *Lj*DII, for use in *L. japonicus* roots (Nadzieja et al., 2018). Compared to the sensor developed in Arabidopsis, we substituted the *L. japonicus* ubiquitin promoter, which shows enhanced expression in legume root tissue relative to the 35S promoter and used a triple repeat of the venus reporter (tYFPnls). To determine if a ratiometric DII was suitable for imaging in *L. japonicus*, we coupled *Lj*DII with an auxin insensitive *Lj*mDII (Liao et al., 2015) fused to NLS-DsRed to create LjR2D2. Both expression units contained *LjUbi* promoter. In transgenic hairy roots both LjR2D2 sensors localized in the nucleus (Figure 3A). Root hair cells of untreated control roots, appeared to have lower tYFP/DsRed fluorescence intensity ratio compared to epidermal non root hair cells (atrichoblasts) suggesting that auxin level in root hairs is elevated compared to other epidermal cells. We extracted tYFP and DsRed fluorescence intensities from 30 root hair and atrichoblasts and calculated the tYFP/DsRed ratio (Figure 3C). Root hair cells showed relatively lower tYFP fluorescence suggesting higher auxin levels compared to atrichoblasts. When treated with IAA, we observed quickly declining YFP fluorescence (Figures 3A,B,D). DsRed fluorescence declined at a much slower rate (Figure 3D).

### Auxin Signaling Reporter

Auxin binds to the nuclear localized receptor TIR1/AFB in SKP1-CUL1-F-box (SCF) ubiquitin ligase complexes (Dharmasiri et al., 2005; Kepinski and Leyser, 2005). The binding results in recruiting auxin/indole acetic acid proteins (Aux/IAAs) into this complex (Gray et al., 2001) and subsequent ubiquitin-mediated degradation of Aux/IAAs. De-repressed auxin response factors (ARFs), may then bind auxin response elements (AuxREs) in gene regulatory elements. Tandem repeats of AuxRE together with a minimal 35S cauliflower mosaic virus (CaMV) promoter constitute the synthetic auxin response promoter DR5 (Liu et al., 1994; Ulmasov et al., 1997).

**Figure 4:**
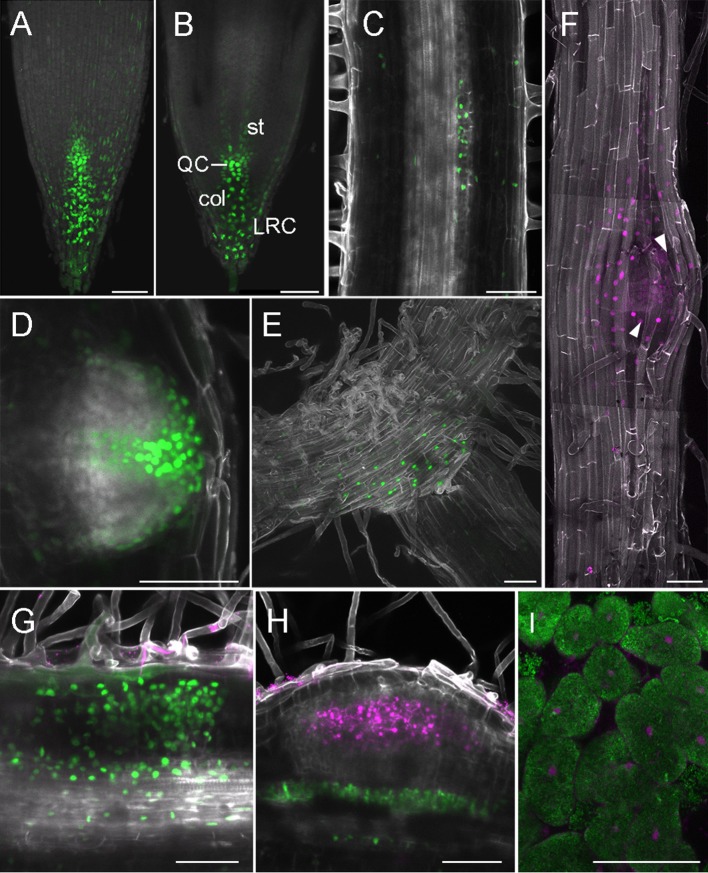
DR5 activity in root and nodule development. **(A–H)** Whole mounts, cleared; **(I)** vibratome section. **(A)** Projection of RAM, **(B)** Confocal section dissecting middle of the root, **(C)** LR at stage Ib/II, **(D)** Emerging LR, **(E)** Base of LR, **(F)** Tile scan of LR breaching epidermis, arrowheads—delaminating epidermal cells, **(G)** Nodule primordium at 6 dpi, **(H)** Nodule with no cortical DR5 activation, **(I)** Section through 14 dpi nodule, inner cells containing symbiosomes. **(A–E,G,H)** DR5-tYFPnls, **(F,I)** DR5-mCherry. **(G,H)** DsRed labeled *M. loti* MAFF303099, **(I)** GFP labeled *M. loti* R7A. Scale bars 50 μm. col, columella; st, stele; LRC, lateral root cap; QC, quiescent center.

In legumes, the DR5 auxin response reporter aided identification of auxin signaling in developing lateral roots (Herrbach et al., 2014) and nodules (Suzaki et al., 2012). The DR5 activity signature displays subtle differences in *L. japonicus* and *M. truncatula*, being expressed in a wider region surrounding individual ITs in *M. truncatula* (Breakspear et al., 2014; Nadzieja et al., 2018). Here, we show that DR5:tYFPnls activity in the *L. japonicus* root apical meristem (RAM) most intensely marks the area of initials, QC, columella and outer root cap cells (Figures 4A,B), which is consistent with patterns described in *A. thaliana* (Blilou et al., 2005). We detected auxin signaling at stage Ib/II of LR formation (first anticlinal cell divisions, Figure 4C), that later organized into a pattern resembling a fully formed RAM (Figure 4D). At the stage of breaching the epidermis (Figure 4F) the auxin signaling zone extends to a subset of epidermal cells with its maxima in the delaminating cells. Activation of DR5 was also observed at the base of elongated lateral roots (Figure 4E).

Consistent with previous observations (Suzaki et al., 2012), in rhizobia inoculated roots, most nodule primordia and nodules showed strong DR5 activation in the root cortex (Figure 4G). Additionally, auxin signaling in cells adjacent to vasculature under sites of infection, was identified. Occasionally, we recorded infected nodules with no DR5 activity within the cortex, but still showing auxin signaling below the infection (Figure 4H), This is consistent with previous reports of DR5 activity being reduced in an autoregulation-dependent manner (Suzaki et al., 2012). We observed DR5 activation in cells containing symbiosomes in sectioned nodules at 14 dpi (Figure 4I).

### Simultaneous Imaging of Auxin and Cytokinin Responses

**Figure 5:**
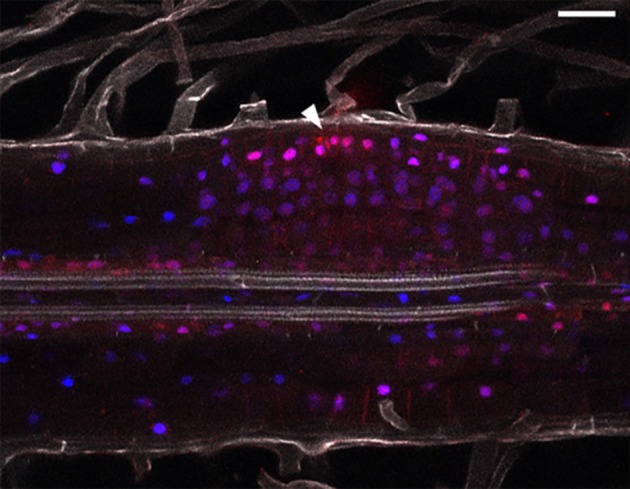
Combined auxin and cytokinin response imaging in *L. japonicus* 5 dpi nodule primordium. Cleared whole mount root. Arrowhead depicts site of epidermal infection. Blue—TCSn:tYFP; red—DR5:mCherry and DsRed labeled rhizobia; gray—autofluorescence. Scale bar 50 μm.

Combining hormonal reporters in the same root might improve understanding of auxin/cytokinin interplay (Fisher et al., 2018). We therefore cloned DR5:mCherry-NLS and TCSn:YFP-NLS into the same expression vector. We observed, that both DR5 and TCSn mark area of cell divisions, but their relative intensities differ (Figure 5). DR5 activity appears higher in cells close to the infection thread (Figure 5, arrowhead) and some neighboring epidermal, atrichoblasts. TCSn maximum intensity is observed in cortical cells below infection sites. Cells situated close to the vasculature show relatively higher DR5 activity.

### Cell Division Reporters

Both auxin and cytokinin regulate the cell cycle and transitions between the phases of the cell cycle are controlled by cyclin-dependent protein kinases (CDKs), whose activity depends on regulatory subunits called cyclins. Transition to mitosis is dependent on B-type cyclins and a cyclin B1;1 (CYCB1;1) marker is commonly used to follow cell cycle progression (Colón-Carmona et al., 1999). The promoter of CycB1;1 is exclusively active in late G2 and early M phase of the cell cycle and translational fusions including the destruction box motif ensure rapid turnover of CYCB1;1 (Colón-Carmona et al., 1999). A *L. japonicus* CycB1;1 promoter and CycB1;1 fragment was used for identification of dividing cells in root meristems and nodule primordia (Soyano et al., 2013).

**Figure 6:**
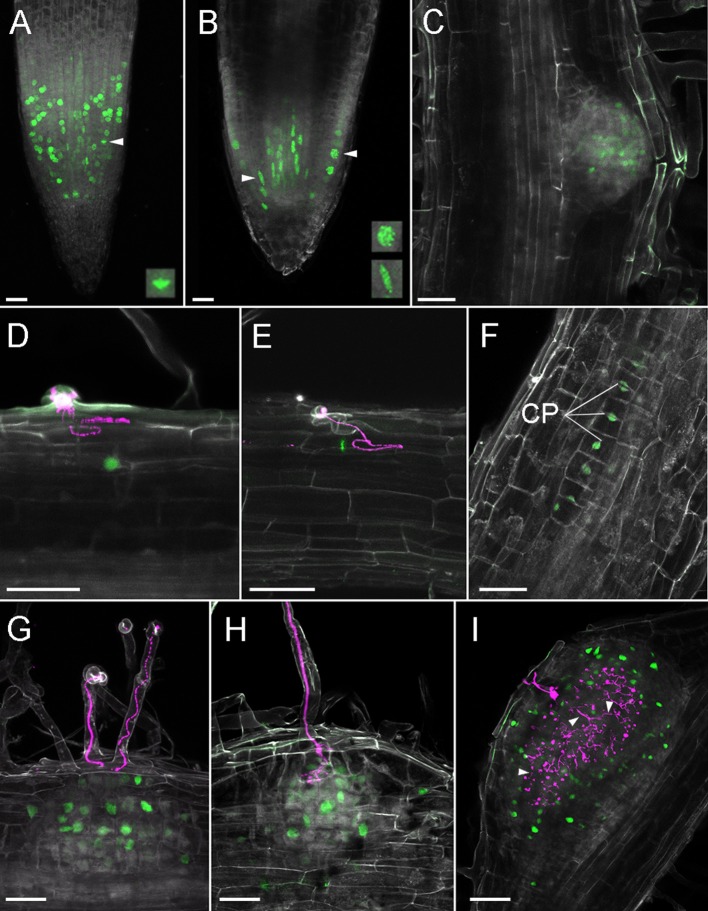
Cell divisions in *L. japonicus* detected using pCYCB1;1::CYCB1^*^-tYFP and confocal microscopy in *L. japonicus* hairy roots. All panels are cleared whole mount roots. **(A)** RAM, intensity projection, arrowhead and box show metaphase nucleus; **(B)** RAM, maximal intensity projection of substack through the middle of the root tip, arrowheads and frames show prophase epidermal (top) and stele (lower) nuclei; **(C)** emerging LR; **(D)** YFP positive nucleus under IT; **(E)** metaphase nucleus under IT; **(F)** 12 cell stage of nodule primordium; **(G)** nodule primordium, ITs do not reach cortex; **(H)** nodule primordium, IT penetrate root cortex; **(I)** nodule with a large network of cortical infection threads, some marked with arrowheads. CP, cell plate; Gray, autofluorescence; green, tYFP fluorescence; magenta, DsRed labeled *M. loti*. Scale bars 50 μm.

In order to visualize cell cycle progression in *L. japonicus* roots, we used an *A. thaliana* cyclin B1;1-based marker (Colón-Carmona et al., 1999). The marker consisted of a cycB1;1 promoter (pCYCB1;1) driving expression of a CYCB1;1-destruction box (CYCB1;1^*^) fused to a reporter protein. For this reporter we created a module containing pCYCB1;1 together with the initial CYCB1;1^*^ coding sequence in order to ease swapping reporter proteins in the modular toolkit. Both the tYFPnls- (Figure 6A) and GUS-based (Figure S2A) reporters showed the characteristic dotted pattern in the RAM as has been observed in Arabidopsis (Colón-Carmona et al., 1999) and *L. japonicus* (Soyano et al., 2013). The cell division zone identified with the construct was clearly confined to the meristem. We also observed cell cycle activation occurring in the stele (Figure 6B, arrowhead, lower frame). We identified a few cells with a strong fluorescence from oblong shaped nuclei (Figure 6A, arrowhead, frame) which is a characteristic of cyclin B1 localization at metaphase (Chen et al., 2008; Yin et al., 2014). A large number of nuclei in the cortical and epidermal layers of the RAM, as well as the stele, showed foci of very strong fluorescence (Figure 6B, arrowhead, top frame). No activity was observed in columella and lateral root cap (Figures 6A,B, Figures S2A,B. We observed variation in intensity of the blue GUS staining in different cells within the cell division zone (Figure S2B), probably due to different cell cycle stages and/or incomplete post-mitotic degradation of the GUS fusion.

During LR organogenesis, the marker was active at stage Ib/II (first anticlinal cell divisions, Herrbach et al., 2014) (Figure S2C). In the enlarging LR meristem, the marker was activated mainly in its central region (Figure S2D). At later stages in emerging lateral roots, activity was localized at the lateral root cap and in the central area proximal to the LR tip (Figure 6C, Figure S2E).

After inoculation with rhizobia we recorded single YFP positive nuclei in the outermost layers of cortex under elongated infection threads (Figures 6D,E). We detected cell divisions within the same layer of cortex (the outermost layer) in a 12-cell, early nodule primordium under an epidermal IT (Figure 6F, IT not shown). In these cells, formation of cell plates could be seen. At later stages of nodule organogenesis we observed cell divisions in additional cortical cell layers (Figures 6G,H). In large nodules, cells divisions occurred around the infected zone and among cortical ITs (Figure 6I).

### Alteration of Auxin Distribution Affects Nodule Emergence Pattern

**Figure 7:**
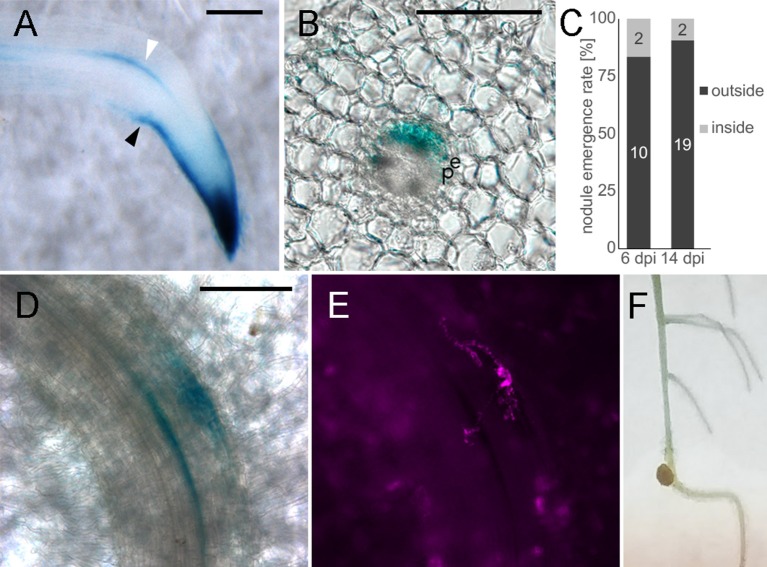
Altered auxin distribution causes biased nodule emergence pattern. Whole mounts **(A,D,E,F)**, transverse vibratome section **(B)**. **(A)** DR5:GUS, 4 h gravitropism stimulation **(B)** 100 μm section of stained root at the gravitropic bend. **(C)** Nodule emergence ratio in gravitropic stimulated roots. *n* = 10 plants for 6 dpi, *n* = 30 for 14 dpi. **(D)** 6 dpi nodule primordium at the gravitropic bend, **(E)** DsRed labeled bacteria, corresponds to D, **(F)** 14 dpi nodule. White arrowhead—DR5 activation in vasculature adjacent cells outside the bend. Black arrowhead—asymmetric DR5 activation in the root tip. e- endodermis, p- pericycle. Scale bars 100 μm.

Given the importance of auxin signaling for rhizobial infection (Breakspear et al., 2014; Nadzieja et al., 2018) and nodule organogenesis (Suzaki et al., 2012) we developed methods to disrupt auxin homeostasis in roots without chemical agonists or antagonists. Gravitropism is well-known to alter root auxin distribution and stimulate lateral root formation (Voß et al., 2015). To observe how gravitropism alters auxin response in *L. japonicus* roots, we grew DR5:GUS stable transgenic plants on agar plates for 7 days. Plates were turned 90° to induce a gravitropic bend before GUS staining of roots attached to the paper (Figure 7, Figure S3). Four hours after inducing gravitropism, an asymmetric auxin response was observed in the root tip, with stronger staining visible on the lower part of the root (Figure S3A). The staining in the epidermis on the lower part of the root extended further from the root tip, compared to the upper side of the root. This was observed for all examined time points (Figure S3). Within 8 h of the stimulus being applied, DR5 was activated near the vasculature on the outer side of the bend (Figure S3, Figure 7A, white arrowhead). This response was localized to the pericycle, as identified by vibratome sectioning (Figure 7B). GUS activity in the pericycle continued to be observed at later time points (Figures S3C–E).

Similar to observations in *A. thaliana* (Voß et al., 2015), lateral root organogenesis predominantly started in the zone of altered auxin distribution on the outside of the bend (Figure S3F). To determine if a gravitropic stimulus is applicable to nodulation studies, we inoculated plants 5 h after gravitropic stimulation and counted the nodules that emerged 5 mm above and below the apex of the bend. Nodules were classified as emerging either inside (lower pericycle auxin) or outside (higher pericycle auxin) of the bend. A higher frequency of nodule emergence on the outside of the bend (Figure 7F) was observed with 19/21 nodules positioned outside the bend (*n* = 30 plants, *p* = 0.0068 in Fisher's exact test with expected 50/50 distribution). To aid in identification of early nodule primordia at the gravitropic bend we replicated the experiment using the DR5:GUS stable line. We carried out staining at 6 dpi of gravitropism stimulated plants (Figures 7D,E) and observed that at this time 10 out of 12 primordia formed on the outside of the bend (n = 10 plants).

## Discussion

Here we present a series of modular tools for visualization of auxin, cytokinin and cell cycle responses in legume roots and nodules and demonstrate their use in *L. japonicus*. Each module is available as a GoldenGate part using standard overhangs. We also identify the fluorescent compatible clearing protocol as a suitable mean for improving imaging depth during studies of root and nodule development in *L. japonicus*. The combination of bright nuclear-localized fluorescent proteins with fixation and clearing helps overcome many of the obstacles to microscopic observations of nodule initiation and development. In our experience, tYFPnls maintains strong fluorescence intensity after fixation and clearing up to several weeks and we recommend it for application in promoter/reporter studies. We found TCSn, DR5, LjDII, and AtCycB1;1 to be robust reporters using this system. The modular nature of our toolkit will allow new components to be added in the future to create new ratiometric or two-color imaging tools such as a 2-color cell cycle progression marker (Yin et al., 2014) or endoreduplication reporters. While GUS detection of cell division is very sensitive, readily observable and can be used for discrete observations of organogenesis, the fluorescent variant of the reporter opens opportunity of prolonged live imaging of cell divisions in developing nodules with 2-photon or light sheet microscopy. Monitoring the shape of the fluorescent signal using the CycB1;1 marker for example presents an interesting future possibility as this may represent distinct phases of the cell cycle.

It was shown that auxin transport is altered upon early stages of the symbiosis (Mathesius et al., 1998; Boot et al., 1999; Pacios-Bras et al., 2003), and that expression patterns of some PIN-encoding genes are distinct in nodule primordia (Huo et al., 2006; Sanko-Sawczenko et al., 2019). Application of gravitropic stimulation alters auxin response localization and has commonly been used in studies of lateral root emergence. Here we show that this technique is also relevant for studies of symbiotic organ formation as nodules preferentially emerge on the outside of a gravitropic bend. This likely results from auxin signaling activation in pericycle and endodermis, which occurs under infection sites in a similar manner to which is observed in gravitropically stimulated roots. The preferential formation of both lateral roots and nodules on the outside of a gravitropic bend illustrates the importance of a localized auxin maxima in both these developmental processes and suggests that these two organogenesis processes share overlapping regulatory mechanisms. Additionally, we noticed that in *L. japonicus* strong activation of DR5 accompanies both developing lateral roots and nodules. In soybean, which also forms determinate type of nodules, DR5 activation appears much more prominent in lateral roots compared to nodules (Turner et al., 2013). This difference might be caused by different naturally occurring AuxREs being employed in nodule and lateral root formation processes in these two species and further analysis is required to determine if this represents a quantitative difference in auxin signaling in the two processes.

Analysis of cytokinin and auxin responses at cellular resolution during later nodule development stages also demonstrated that auxin signaling is activated in the cells containing symbiosomes in mature nodules, while cytokinin signaling appears to be exclusive to infection in the epidermis, cortex and nodules. The DR5 activation in symbiosomes may originate from auxin produced by both the plant as well as the bacteria, as auxin content in nodules can depend on microbial ability to produce IAA (Spaepen et al., 2007).

TCSn activation in nodule development resembles promoter activity of *Log4*, which was identified as the most strongly upregulated cytokinin biosynthesis gene in *L. japonicus* nodulation (Reid et al., 2017). Comparison of TCSn and promoters of individual cytokinin biosynthesis genes or other cytokinin signaling components in a multi-fluorescent reporter construct could be an interesting application of our toolkit.

Our observations support that auxin signaling is associated with both cell division events and infection, whereas cytokinin signaling is inhibitory for infection but required for cell division. Auxin and cytokinin are both involved in the plant cell cycle activation and progression. Expression of a number of cell cycle genes was shown to be upregulated in root hairs during symbiosis (Breakspear et al., 2014) whereas no B-type cyclins, associated with mitosis, were found to be induced. This is consistent with our analysis of the CYCB1-based cell cycle progression marker which marks cell divisions occurring in the cortex during nodule primordia initiation, but was not detected in root hairs. The partial cell cycle activation associated with infection may then result from auxin signaling in the absence of cytokinin.

In conclusion, our modular toolkit for study of auxin, cytokinin and cell cycle progression should enhance our ability to understand root and nodule development in legumes.

## Materials and Methods

### Biological Material and Growth Conditions

*Lotus japonicus* ecotype Gifu was used in all presented experiments. *Mesorhizobium loti* MAFF303099 labeled with DsRed and *M. loti* R7A labeled with GFP were used as a compatible symbionts. Transformed hairy root plants for imaging were grown in large plastic Microboxes (SacO2, Deinze, Belgium) in 4:1 LECA:Vermiculite substrate supplemented with 1/4 strength B&D nutrients (Broughton and Dilworth, 1971). Growth conditions were set to 16/8 h light/dark cycle with 20°C/15°C temperature.

### Cloning and Available Toolkit Components

**Figure 8:**
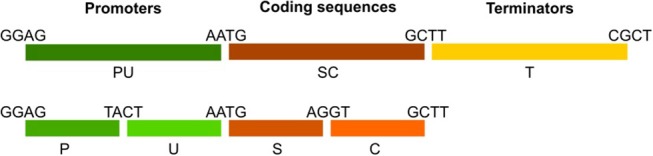
Transcriptional unit assembly and module naming standards. Promoters are available as “PU” or separate “P” and “U” parts. Coding sequences are represented by “SC” modules or separate “S” and “C” parts to enable tagging at N- or C-terminal locations. Terminator modules are termed “T.” Overhangs connecting each part are indicated. Note the PU-SC connection contains the ATG start codon.

The GoldenGate modular cloning system using type IIs restriction enzymes was used for cloning and assembly of desired vectors (Weber et al., 2011). In order to ensure compatibility and exchange of genetic parts, we based our modular toolkit on GoldenGate modules using standardized overhangs (Patron et al., 2015). Module assembly schematic and available modules are shown (Table 1, Figure 8) together with accession numbers for requesting constructs from Lotus Base (lotus.au.dk; Mun et al., 2016). Level 1 constructs were created by combining level 0 modules into expression units containing the promoter (PU) coding sequence (SC) and terminator (T) modules. Level 1 constructs were obtained in reactions of 20 μl containing 100 ng of each level 0 module required for creation of desired construct, 100 ng of target vector, 5 U T4 ligase (Thermo), 2,5 U BsaI (New England BioLabs), 2 μl 10x T4 ligase buffer, 2 μl 10x BSA and water. Goldengate reactions were cycled between 37°C (3 min) and 16°C (4 min) 25 times to favor digestion-ligations reactions respectively.

**Table 1:** GoldenGate cloning modules available in the toolkit.

**Functional units**	**Name**	**Module type**	**5^**′**^/3^**′**^ overhangs**	**References**	**Accession**
Transformation vectors	pIV10-L1	L1 acceptor	GGAG/CGCT		PMC-01391
	pIV10-L2	L2 acceptor	TGCC/GGGA		PMC-01270
Promoters	LjUbi	P	GGAG/TACT		PMC-03530
	LjUbi	PU	GGAG/AATG		PMC-01500
	TCSn			Zürcher et al., 2013	PMC-01331
	DR5			Ulmasov et al., 1997	PMC-01243
	AtCycB1;1^*^			Colón-Carmona et al., 1999	PMC-01241
Coding sequences	DII	U	TACT/AATG	Brunoud et al., 2012	PMC-01269
	mDII			Liao et al., 2015	PMC-03531
	NLS	S	AATG/AGGT		PMC-01303
	GUS	SC	AATG/GCTT		PMC-01722
	tYFP-NLS			Reid et al., 2016	PMC-01484
	mCherry	C	AGGT/GCTT		PMC-01302
	DsRed				PMC-01232
Terminators	35s	T	GCTT/CGCT		PMC-01118
	OCS				PMC-01319

* *Full sequences of all modules are provided in Supplemental File 1. AtCycB1;1 PU module contains promoter and cyclin B1;1 destruction box*.

### Hairy Root Transformation

Prior to hairy root transformation, pIV10-based genetic constructions were transferred from donor *E. coli* to *A. rhizogenes* AR1193 as previously described (Stougaard et al., 1987). Hairy roots were induced according to Hansen et al. (1989).

### Fluorescence Compatible Clearing

The procedure of clearing optimized for *Pisum sativum* nodule was used essentially as previously described (Warner et al., 2014) with the following modifications for *L. japonicus* roots. Fluorescence positive samples were harvested and fixed overnight in 4% p-formaldehyde, 80mM PIPES pH7 solution. Then, rinsed 3 times for 5 min in 80 mM PIPES pH7. Prior to imaging, samples were incubated for 10 days in clearing solution containing 6M urea, 30% v/v glycerol, 0,01% Triton X-100, 40 mM PIPES pH 7.

### Physical Sectioning of Roots and Nodules

Roots and nodules were mounted in molten 3% agarose. To conserve fluorescence, agarose was precooled to 50°C. Solidified blocks were then excised and cut using Leica VT1000S vibratome. Sections of 60–100 μm were cut and mounted onto microscopic slides.

### GUS Staining

Plant material was submerged in GUS staining solution (0,5 mg/ml 5-bromo-4-chloro-3-indolyl-beta-D-glucuronic acid, 100 mM NaPO_4_, 10 mM EDTA, 1 mM Potassium Ferricyanide, 1 mM Potassium Ferrocyanide, 0,1% Triton X-100, pH 7). Vacuum infiltration was applied for 10 min. Samples were stained in darkness at 21°C for several hours or overnight. Staining was stopped by removing the staining solution and rinsing the samples with 70% ethanol.

### Gravitropic Stimulation

Prior to 90° turn in root growth direction, plants were grown for 7 days on upright agar plates supplemented with 1/4 strength B&D nutrients. For monitoring of DR5 response upon gravitropic stimulation, plants were harvested and examined for β-glucuronidase activity at 4, 8, 16, 24, and 42 h after applying the stimulus. The roots were not detached from filter paper in order to maintain the shape of gravitropic bends during staining. For nodulation assays, plants were inoculated after 7 days growth on ¼ B&D medium and root growth direction was changed 5 h after inoculation. Nodules emerging outside and inside the gravitropic bend were distinguished. Statistical significance of the bias in nodule position was determined by Fisher's exact test, where expected distribution of nodule emergence inside/outside the bend equaled 50/50.

### Microscopy and Image Processing

Confocal microscopy was performed with Zeiss LSM510 and Zeiss LSM780 microscopes. For LSM780, following excitation/emission [nm] settings were used: (i) autofluorescence of cell components 405/420–505, (ii) GFP 488/490–550, (iii) YFP 514/517–560, (iv) mCherry 561/570–700, (v) DsRed 561/580–660. For LSM510: (i) YFP 514/530–600, (ii) DsRed 543/560–615. Images of larger size samples were constructed by acquisition of smaller images and stitching them together (tile scans). For LjR2D2, 18-colors look up table (LUT) was applied to ease identification of fluorescence gradients. GUS and non-confocal DsRed fluorescence images were obtained using Zeiss Axioplan 2. Zeiss ZEN and Fiji ImageJ (Schindelin et al., 2012) were used for image processing and extraction of fluorescence intensities values.

## Data Availability

All datasets generated for this study are included in the manuscript and/or the Supplementary Files.

## Author Contributions

MN, JS, and DR conceived the research plan. MN conducted experiments and microscopy. MN prepared the figures and wrote the manuscript together with JS and DR.

### Conflict of Interest Statement

The authors declare that the research was conducted in the absence of any commercial or financial relationships that could be construed as a potential conflict of interest.

## Supplementary Material

Figure S1 Impact of fluorescence compatible clearing on imaging depth in *L. japonicus* root. Roots expressing *Lj*Ubi:tYFPnls, confocal image of median longitudinal section **(A)** uncleared root **(B)** Root cleared for 10 days. Scale bars 100 μm. Resolution obtained in XY, and YZ axes are shown.

Figure S2 GUS-dependent identification of cell divisions in *L. japonicus* roots. pCYCB1;1::CYCB1^*^-GUS construct was expressed in *L. japonicus* hairy roots. **(A,B)** RAM, staining confined to the cell division zone **(C)** LR emergence from the pericycle **(D)** stage Ib/II LR, **(D)** staining in the central region of the LR, **(E)** more uniform staining in the LR; **(F–I)** nodule primordia, cell divisions localized in the cortex **(J,K)** mature nodule, marker activity observed inside the nodule. **(A,E,J)** dark field; **(B,F)** phase contrast; **(C,D,H)** bright field; **(G,I,K)** DsRed fluorescence corresponding to **(F,H,J)**. Scale bars indicate 50 μm.

Figure S3 DR5:GUS activity upon gravitropic stimulation in *L. japonicus* roots. GUS staining at **(A)** 4 h, **(B)** 8 h (shown in Figure 7A), **(C)** 16 h, **(D)** 24 h, **(E,F)** 48 h after application of the stimulus. White arrowhead—asymmetric, vasculature adjacent DR5 activation outside the bend, black arrowheads—asymmetric DR5 activity in the root tip. Box in F showing emerging LR at the bending site. Scale bars 200 μm.

Video S1 Fluorescent compatible clearing in the root outside root apical meristem Lj DII transgenic root. Green—tYFP.

Video S2 Application of fluorescent compatible clearing for improving visualization of cortical infection threads. 6 dpi nodule primordium. Gray—autofluorescence of root cell walls, magenta—DsRed labeled *M. loti*.

File S1 ImageJ macro and sequences of GoldenGate modules.
