# Supplementary material for: A Toolkit for High Resolution Imaging of Cell Division and Phytohormone Signaling in Legume Roots and Root Nodules
Source: Front Plant Sci. 2019 Aug 2;10:1000. doi: 10.3389/fpls.2019.01000 (PMC6688427; doi:10.3389/fpls.2019.01000)
Supplement: Figure S1 — Impact of fluorescence compatible clearing on imaging depth in L. japonicus root. Roots expressing LjUbi:tYFPnls, confocal image of median longitudinal section (A) uncleared root (B) Root cleared for 10 days. Scale bars 100 μm. Resolution obtained in XY, and YZ axes are shown. [file Table_1.DOCX]

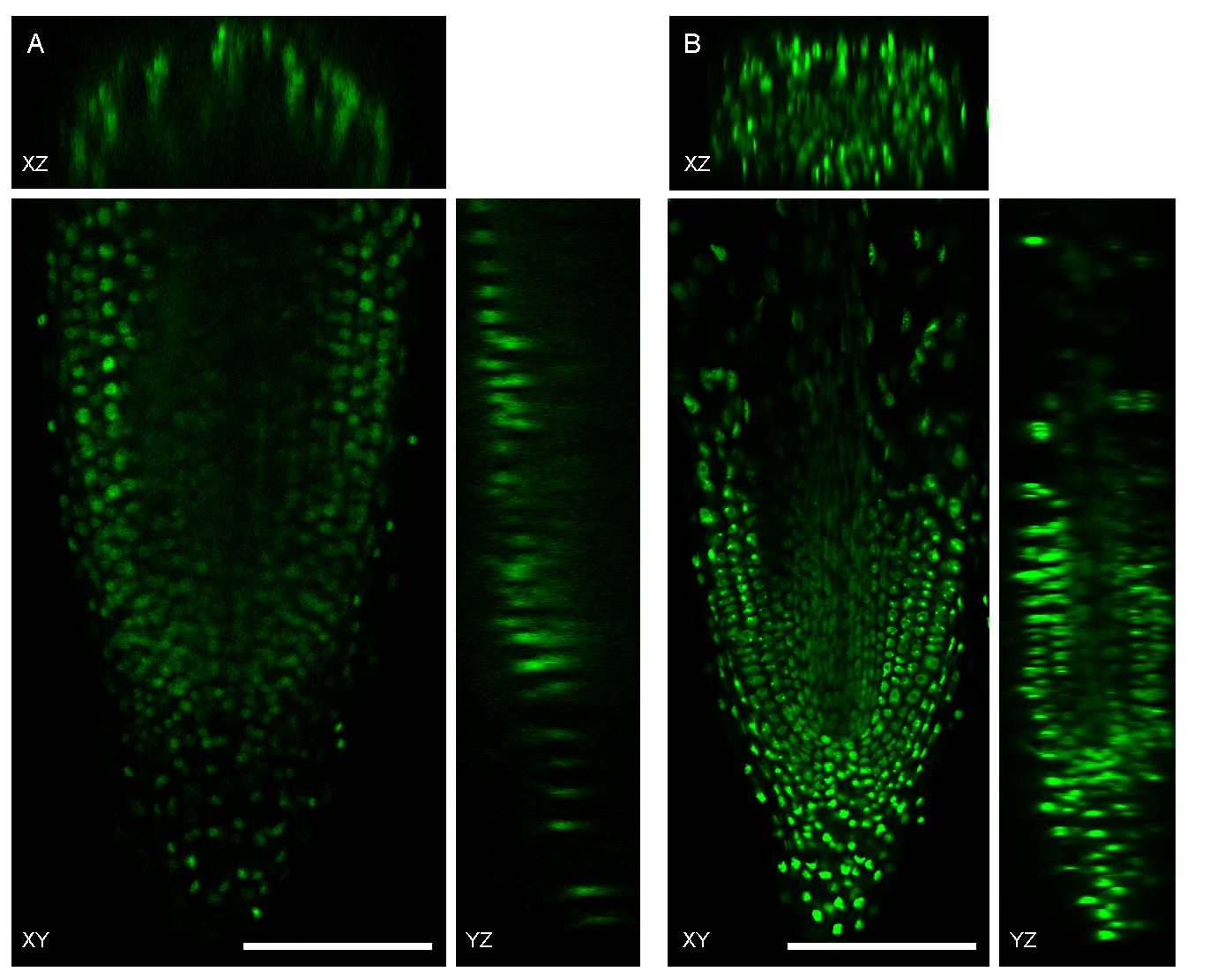


Figure S1. Impact of fluorescence compatible clearing on imaging depth in L. japonicus

root. Roots expressing LjUbi:tYFPnls, confocal image of median longitudinal section A)

uncleared root B) Root cleared for 10 days. Scale bars 100 μm. Resolution obtained in XY,

and YZ axes are shown.


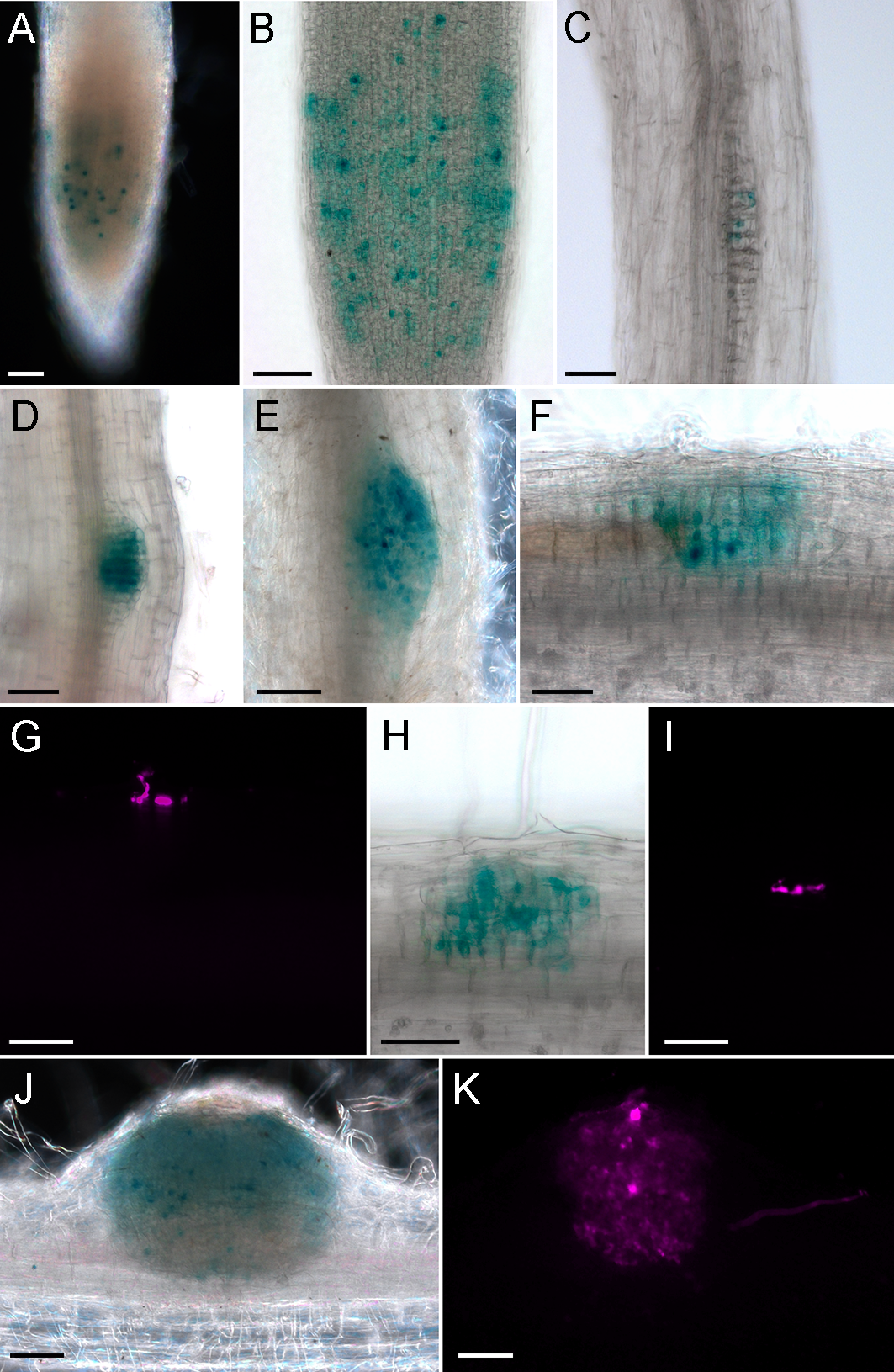


Figure S2. GUS-dependent identification of cell divisions in L. japonicus roots.

pCYCB1;1::CYCB1*-GUS construct was expressed in L. japonicus hairy roots. AB) RAM,

staining confined to the cell division zone C) LR emergence from the pericycle D) stage

Ib/II LR, D) staining in the central region of the LR, E) more uniform staining in the LR; F-I)

nodule primordia, cell divisions localized in the cortex J-K) mature nodule, marker activity

observed inside the nodule. A,E,J) dark field; B,F) phase contrast; C,D,H) bright field;

G,I,K) DsRed fluorescence corresponding to F,H,J. Scale bars indicate 50 μm.


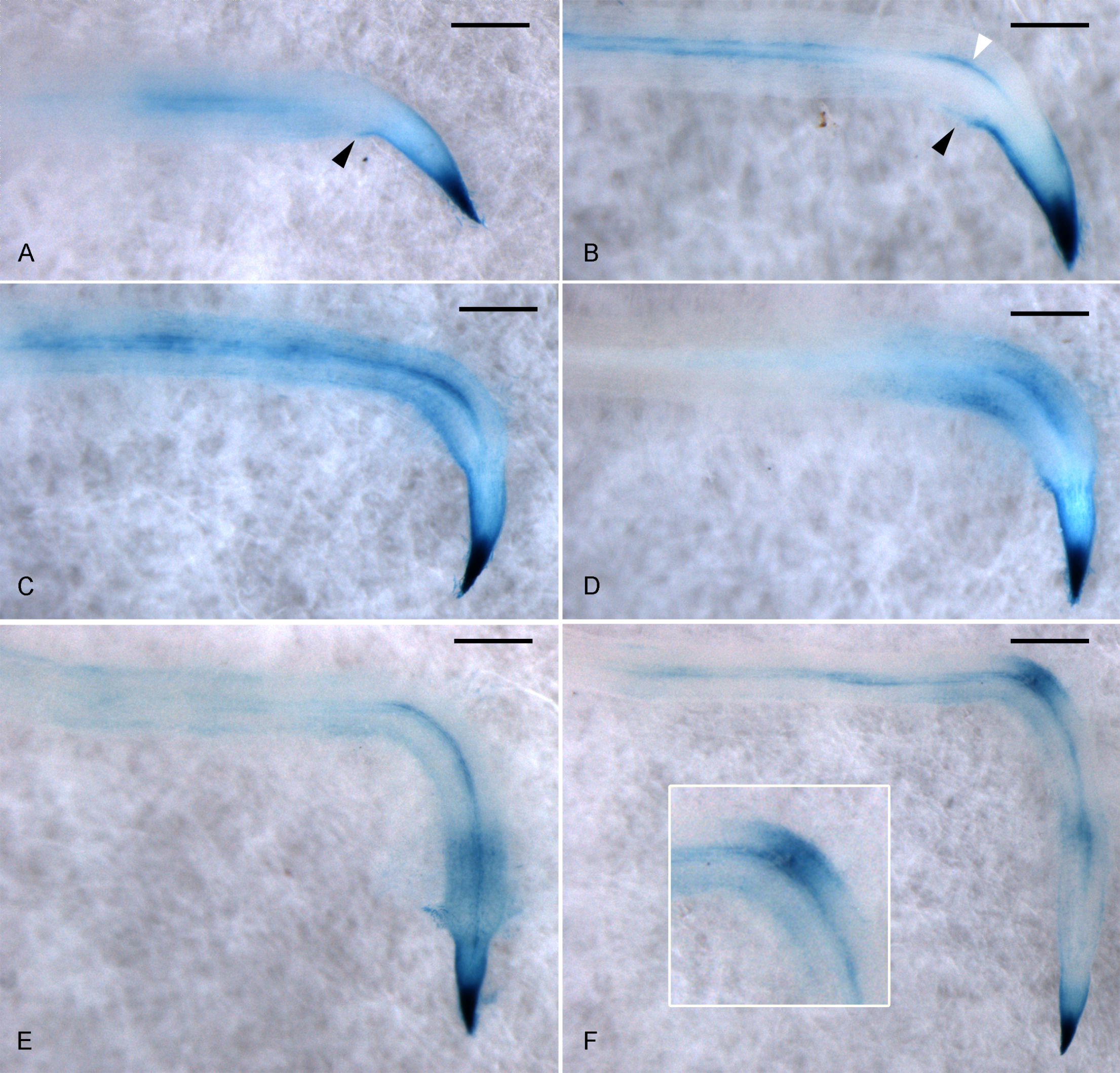


Figure S3. DR5:GUS activity upon gravitropic stimulation in L. japonicus roots. GUS

staining at A) 4h, B) 8h (shown in Fig. 7A), C) 16h, D) 24h, E and F) 48h after application of the stimulus. White arrowhead – asymmetric, vasculature adjacent DR5 activation outside the bend, black arrowheads – asymmetric DR5 activity in the root tip. Box in F showing emerging LR at the bending site. Scale bars 200 μm.
