# Supplementary material for: A Toolkit for High Resolution Imaging of Cell Division and Phytohormone Signaling in Legume Roots and Root Nodules
Source: Front Plant Sci. 2019 Aug 2;10:1000. doi: 10.3389/fpls.2019.01000 (PMC6688427; doi:10.3389/fpls.2019.01000)
Supplement: File S1 — ImageJ macro and sequences of GoldenGate modules. [file Data_Sheet_1.docx]

Supplemental file.

The following macro in Fiji ImageJ was created for generating ratiometric images of LjR2D2 sensor:

>

selectWindow("R2D2_timecourse.tif");
run("32-bit");
run("Split Channels");
saveAs("Tiff", "/*Users/PM/Desktop/DsRed_R2D2_timecourse.tif*");
selectWindow("C1-R2D2_timecourse.tif");
saveAs("Tiff", "*/Users/PM/Desktop/tYFP_R2D2_timecourse.tif*");
selectWindow("*DsRed_R2D2_timecourse.tif*");
run("Gaussian Blur...", "sigma=0.70 stack");
selectWindow("*tYFP_R2D2_timecourse.tif*");
run("Gaussian Blur...", "sigma=0.70 stack");
selectWindow("*DsRed_R2D2_timecourse.tif*");
//run("Brightness/Contrast...");
run("Enhance Contrast", "saturated=0.35");
run("Close");
selectWindow("*tYFP_R2D2_timecourse.tif*");
//run("Brightness/Contrast...");
run("Enhance Contrast", "saturated=0.35");
run("Close");
imageCalculator("Divide create 32-bit stack", "*DsRed_R2D2_timecourse.tif*","*tYFP_R2D2_timecourse.tif*");
selectWindow("*Result of DsRed_R2D2_timecourse.tif*");
selectWindow("*DsRed_R2D2_timecourse.tif*");
selectWindow("*Result of DsRed_R2D2_timecourse.tif*");
run("16 Colors");
saveAs("Tiff", "*/Users/PM/Desktop/R2D2_timecourse_ratio.tif*");

<

Underline indicates original time lapse in *.tif* format and *italic* marks selected access path and filenames.

Golden gate modules presented in this study.

Sequences include bordering *BsaI* sites

PMC-01097

P-LjUbi

GGTCTCAGGAGGGAGAGAGGATTTTGAGGAAATAATTAATTGAATTACTTGTATTATTGATAAAGTAATTTAGATAAGTTGTTAGTACAACTTATTGCAACTATGGATAGACAAAAATCACTTATTTTAAGGGGAGAAGTATATAACAACTTATTATAAAATTTCTGAGCAGCCGGCCTCCTCCAATCAATCATAGAGAGTGGAGCCCATTCTGGAAAACCAAGGAACCCCCAACTTGCACTGGTGCGGTGGCCCAATTCAAAAAAAACGGGGCCCAGCAAAGTAACGCCGTTGTAACGATTTATCAATCCAAACTCAAAAGGCGGCGAGATGCGTTTAACTCCGTGAAATTAACAAACCGCCAACAACTTGCAATTTGCAACTACCGTTTCCAAGAAGAACTCAACCACACAACGTATCCTATCCCAAACCACACGCAACTAGTGACGCGTCATAAGGACACGTGTCACAATTTGACTGGTTAATAATTTCACCGCTTTTGCTATAAATTACCTCCAATCCCCTTAGCTTCTTCACAATTCAGTTCCCAACCCTAACAATTCTTGTTCATATCGCTTCTCTCTACTTTCAAGGTATGATCCAATTTCTCTCTTCTTTCTCTGTAATCCTTTCGTTGAGTTTTGTTTCCGATCAATCATAGGTAGTTTTCTTGTTTCGAAGCATGAGATCTAGGAATTTTTTGTGATTTTCCAAAATTGAGATCGGTTTGAAATTGAATTTTACAGCTTGAATCTCAGATCTTGTTTTATCAATGTTTTCGATGGCTTGCGATGTAGATCTATGATAATTGTGGTTCAGTTTTGTTAGGAATCGATTTCGGTTTAGCAATTGCAGATTAATTAGGGTTTCCAATTGAATTCTTCAGATCCGTTATGGAATTATGTCAAATAATTTATTCAAATTGGAAATTATTGTTAGATCCACTCTTAATCTGTTTGATCCAAGCTTCAATTAGGGTTTTCACTTGTTTCAATTTCTTGTTATGGATTCTGATTTATCTGTTGATGTTAGATCCACTCTTAATCTGTTTGATCCAAGCGTTAATTAGGGTTTTCACTTGTTTCAATTTCGTGTGTTGGATTCTGATTTATCTGTTGTTGATGTGATTACAGTACTTGAGACC

PMC-01500

PU-LjUbi

GGTCTCAGGAGGGAGAGAGGATTTTGAGGAAATAATTAATTGAATTACTTGTATTATTGATAAAGTAATTTAGATAAGTTGTTAGTACAACTTATTGCAACTATGGATAGACAAAAATCACTTATTTTAAGGGGAGAAGTATATAACAACTTATTATAAAATTTCTGAGCAGCCGGCCTCCTCCAATCAATCATAGAGAGTGGAGCCCATTCTGGAAAACCAAGGAACCCCCAACTTGCACTGGTGCGGTGGCCCAATTCAAAAAAAACGGGGCCCAGCAAAGTAACGCCGTTGTAACGATTTATCAATCCAAACTCAAAAGGCGGCGAGATGCGTTTAACTCCGTGAAATTAACAAACCGCCAACAACTTGCAATTTGCAACTACCGTTTCCAAGAAGAACTCAACCACACAACGTATCCTATCCCAAACCACACGCAACTAGTGACGCGTCATAAGGACACGTGTCACAATTTGACTGGTTAATAATTTCACCGCTTTTGCTATAAATTACCTCCAATCCCCTTAGCTTCTTCACAATTCAGTTCCCAACCCTAACAATTCTTGTTCATATCGCTTCTCTCTACTTTCAAGGTATGATCCAATTTCTCTCTTCTTTCTCTGTAATCCTTTCGTTGAGTTTTGTTTCCGATCAATCATAGGTAGTTTTCTTGTTTCGAAGCATGAGATCTAGGAATTTTTTGTGATTTTCCAAAATTGAGATCGGTTTGAAATTGAATTTTACAGCTTGAATCTCAGATCTTGTTTTATCAATGTTTTCGATGGCTTGCGATGTAGATCTATGATAATTGTGGTTCAGTTTTGTTAGGAATCGATTTCGGTTTAGCAATTGCAGATTAATTAGGGTTTCCAATTGAATTCTTCAGATCCGTTATGGAATTATGTCAAATAATTTATTCAAATTGGAAATTATTGTTAGATCCACTCTTAATCTGTTTGATCCAAGCTTCAATTAGGGTTTTCACTTGTTTCAATTTCTTGTTATGGATTCTGATTTATCTGTTGATGTTAGATCCACTCTTAATCTGTTTGATCCAAGCGTTAATTAGGGTTTTCACTTGTTTCAATTTCGTGTGTTGGATTCTGATTTATCTGTTGTTGATGTGATTACAGAATGTGAGACC

PMC-01243

PU-DR5

GGTCTCAGGAGACAAAAGGGAGACAAAAGGGAGACAAAAGGGAGACAAAAGGGAGACAAAAGGGAGACAAAAGGGAGACAAAAGGGAGACAAAAGGGAGACAAAAGGGGGCAGGCCTCGATAAGCTTGATATCGAATTAATTCCTGCAGCCCCGCAAGACCCTTCCTCTATATAAGGAAGTTCATTTCATTTGGAGAGGTATTTTTACAACAATTACCAACAACAACAAACAACAAACAACATTACAATTACTATTTACAATTACAATTACAGGGGATCGATCCAAGGAGATATAACAATGTGAGACC

PMC-01331

PU-TCSn

GGTCTCAGGAGCTAAAAGCTTGACTAGTCAAAGATCTTTAAAAGATTTTGAAAGATCTCTCCAAAATCCTTTCAAAGATCTTTAAAAGATTTATAAAAATCTTTGCAAAATCCAACCAAAGATTTTGTAAAGATTTTGCAAGATCCGATCAAAATCTTTAGCTAGTCAAAGATCTTTAAAAGATTTTGAAAGATCTCTCCAAAATCCTTTCAAAGATCTTTAAAAGATTTATAAAAATCTTTGCAAAATCCAACCAAAGATTTTGTAAAGATTTTGCAAGATCCGATCAAAATCTTTAGCTAGCCAAGACCCTTCCTCTATATAAGGAAGTTCATTTCATTTGGAGAGGATCTGTATTTTTACAACAATTACCAACAACAACAAACAACAAACAACATTACAATTACTATTTACAATTACAATTACAGGGGATCGATCCAAGGAGATATAACAAATGTGAGACC

PMC-01269

U-DII

GGTCTCATACTGTATTTTTACAACAATTACCAACAACAACAAACAACAAACAACATTACAATTACTATTTACAATTACATGAAACAAAAAAGCTCGACCAAAGAAACATCATTCCTTTCCAATAACAGGGTTGAGGTAGCTCCAGTGGTGGGATGGCCGCCGGTGAGATCATCCCGGAGAGGAATGTGAGACC

PMC-03531

U-mDII

GGTCTCATACTGTATTTTTACAACAATTACCAACAACAACAAACAACAAACAACATTACAATTACTATTTACAATTACATGAAACAAAAAAGCTCGACCAAAGAAACATCATTCCTTTCCAATAACAGGGTTGAGGTAGCTCCAGTGGTGGGATGGCtGCCGGTGAGATCATCCCGGAGAGGAATGTGAGACC

PMC-01303

S-NLS

GGTCTCAAATGCCTAAGAAGAAGAGAAAGGTTGGAGGTTGAGACC

PMC-01722

SC-GUS

GGTCTCAAATGGGTCAGTCCCTTATGTTACGTCCTGTAGAAACCCCAACCCGTGAAATCAAAAAACTCGACGGCCTGTGGGCATTCAGTCTGGATCGCGAAAACTGTGGAATTGATCAGCGTTGGTGGGAAAGCGCGTTACAAGAAAGCCGGGCAATTGCTGTGCCAGGCAGTTTTAACGATCAGTTCGCCGATGCAGATATTCGTAATTATGCGGGCAACGTCTGGTATCAGCGCGAAGTCTTTATACCGAAAGGTAAGTCTTACTCTCTCTTTTTTGGTCTGTATTTTTAATTTTTTGAAGTATACTATTTGTACTGACGCTAATAATCTTTTTTCAGGTTGGGCAGGCCAGCGTATCGTGCTGCGTTTCGATGCGGTCACTCATTACGGCAAAGTGTGGGTCAATAATCAGGAAGTGATGGAGCATCAGGGCGGCTATACGCCATTTGAAGCCGATGTCACGCCGTATGTTATTGCCGGGAAAAGTGTACGTATCACCGTTTGTGTGAACAACGAACTGAACTGGCAGACTATCCCGCCGGGAATGGTGATTACCGACGAAAACGGCAAGAAAAAGCAGTCTTACTTCCATGATTTCTTTAACTATGCCGGAATCCATCGCAGCGTAATGCTCTACACCACGCCGAACACCTGGGTGGACGATATCACCGTGGTGACGCATGTCGCGCAAGACTGTAACCACGCGTCTGTTGACTGGCAGGTACTTCATGCTTCAACGTGTAACTTAAGAGATACTGTGTGAAATTTTATATTTCCATACATTTGCTTGACCTTTGCTTTTTGTCAATTTTTTTCCCCTTACAGGTGGTGGCCAATGGTGATGTCAGCGTTGAACTGCGTGATGCGGATCAACAGGTGGTTGCAACTGGACAAGGCACTAGCGGGACTTTGCAAGTGGTGAATCCGCACCTCTGGCAACCGGGTGAAGGTTATCTCTATGAACTGTGCGTCACAGCCAAAAGCCAGACAGAGTGTGATATCTACCCGCTTCGCGTCGGCATCCGGTCAGTGGCAGTGAAGGGCGAACAGTTCCTGATTAACCACAAACCGTTCTACTTTACTGGCTTTGGTCGTCATGAAGATGCGGACTTGCGTGGCAAAGGATTCGATAACGTGCTGATGGTGCACGACCACGCATTAATGGACTGGATTGGGGCCAACTCCTACCGTACCTCGCATTACCCTTACGCTGAAGAGATGCTCGACTGGGCAGATGAACATGGCATCGTGGTGATTGATGAAACTGCTGCTGTCGGCTTTAACCTCTCTTTAGGCATTGGTTTCGAAGCGGGCAACAAGCCGAAAGAACTGTACAGCGAAGAGGCAGTCAACGGGGAAACTCAGCAAGCGCACTTACAGGCGATTAAAGAGCTGATAGCGCGTGACAAAAACCACCCAAGCGTGGTGATGTGGAGTATTGCCAACGAACCGGATACCCGTCCGCAAGGTGCACGGGAATATTTCGCGCCACTGGCGGAAGCAACGCGTAAACTCGACCCGACGCGTCCGATCACCTGCGTCAATGTAATGTTCTGCGACGCTCACACCGATACCATCAGCGATCTCTTTGATGTGCTGTGCCTGAACCGTTATTACGGATGGTATGTCCAAAGCGGCGATTTGGAAACGGCAGAGAAGGTACTGGAAAAAGAACTTCTGGCCTGGCAGGAGAAACTGCATCAGCCGATTATCATCACCGAATACGGCGTGGATACGTTAGCCGGGCTGCACTCAATGTACACCGACATGTGGAGTGAAGAGTATCAGTGTGCATGGCTGGATATGTATCACCGCGTCTTTGATCGCGTCAGCGCCGTCGTCGGTGAACAGGTATGGAATTTCGCCGATTTTGCGACCTCGCAAGGCATATTGCGCGTTGGCGGTAACAAGAAAGGGATCTTCACTCGCGACCGCAAACCGAAGTCGGCGGCTTTTCTGCTGCAAAAACGCTGGACTGGCATGAACTTCGGTGAAAAACCGCAGCAGGGAGGCAAACAATGAGCTTTGAGACC

PMC-01484

SC-tYFP

GGTCTCAAATGGTGAGCAAGGGCGAGGAGCTGTTCACCGGGGTGGTGCCCATCCTGGTCGAGCTGGACGGCGACGTAAACGGCCACAAGTTCAGCGTGTCCGGCGAGGGCGAGGGCGATGCCACCTACGGCAAGCTGACCCTGAAGCTGATCTGCACCACCGGCAAGCTGCCCGTGCCCTGGCCCACCCTCGTGACCACCCTGGGCTACGGCCTGCAGTGCTTCGCCCGCTACCCCGACCACATGAAGCAGCACGACTTCTTCAAGTCCGCCATGCCCGAAGGCTACGTCCAGGAGCGCACCATCTTCTTCAAGGACGACGGCAACTACAAGACCCGCGCCGAGGTGAAGTTCGAGGGCGACACCCTGGTGAACCGCATCGAGCTGAAGGGCATCGACTTCAAGGAGGACGGCAACATCCTGGGGCACAAGCTGGAGTACAACTACAACAGCCACAACGTCTATATCACCGCCGACAAGCAGAAGAACGGCATCAAGGCCAACTTCAAGATCCGCCACAACATCGAGGACGGCGGCGTGCAGCTCGCCGACCACTACCAGCAGAACACCCCCATCGGCGACGGCCCCGTGCTGCTGCCCGACAACCACTACCTGAGCTACCAGTCCGCCCTGAGCAAAGACCCCAACGAGAAGCGCGATCACATGGTCCTGCTGGAGTTCGTGACCGCCGCCGGGATCACTCTCGGCATGGACGAGGCAGCTAGATCCACCATGGTGAGCAAGGGCGAGGAGCTGTTCACCGGGGTGGTGCCCATCCTGGTCGAGCTGGACGGCGACGTAAACGGCCACAAGTTCAGCGTGTCCGGCGAGGGCGAGGGCGATGCCACCTACGGCAAGCTGACCCTGAAGCTGATCTGCACCACCGGCAAGCTGCCCGTGCCCTGGCCCACCCTCGTGACCACCCTGGGCTACGGCCTGCAGTGCTTCGCCCGCTACCCCGACCACATGAAGCAGCACGACTTCTTCAAGTCCGCCATGCCCGAAGGCTACGTCCAGGAGCGCACCATCTTCTTCAAGGACGACGGCAACTACAAGACCCGCGCCGAGGTGAAGTTCGAGGGCGACACCCTGGTGAACCGCATCGAGCTGAAGGGCATCGACTTCAAGGAGGACGGCAACATCCTGGGGCACAAGCTGGAGTACAACTACAACAGCCACAACGTCTATATCACCGCCGACAAGCAGAAGAACGGCATCAAGGCCAACTTCAAGATCCGCCACAACATCGAGGACGGCGGCGTGCAGCTCGCCGACCACTACCAGCAGAACACCCCCATCGGCGACGGCCCCGTGCTGCTGCCCGACAACCACTACCTGAGCTACCAGTCCGCCCTGAGCAAAGACCCCAACGAGAAGCGCGATCACATGGTCCTGCTGGAGTTCGTGACCGCCGCCGGGATCACTCTCGGCATGGACGAGGCAGCTAGATCCACCATGGTGAGCAAGGGCGAGGAGCTGTTCACCGGGGTGGTGCCCATCCTGGTCGAGCTGGACGGCGACGTAAACGGCCACAAGTTCAGCGTGTCCGGCGAGGGCGAGGGCGATGCCACCTACGGCAAGCTGACCCTGAAGCTGATCTGCACCACCGGCAAGCTGCCCGTGCCCTGGCCCACCCTCGTGACCACCCTGGGCTACGGCCTGCAGTGCTTCGCCCGCTACCCCGACCACATGAAGCAGCACGACTTCTTCAAGTCCGCCATGCCCGAAGGCTACGTCCAGGAGCGCACCATCTTCTTCAAGGACGACGGCAACTACAAGACCCGCGCCGAGGTGAAGTTCGAGGGCGACACCCTGGTGAACCGCATCGAGCTGAAGGGCATCGACTTCAAGGAGGACGGCAACATCCTGGGGCACAAGCTGGAGTACAACTACAACAGCCACAACGTCTATATCACCGCCGACAAGCAGAAGAACGGCATCAAGGCCAACTTCAAGATCCGCCACAACATCGAGGACGGCGGCGTGCAGCTCGCCGACCACTACCAGCAGAACACCCCCATCGGCGACGGCCCCGTGCTGCTGCCCGACAACCACTACCTGAGCTACCAGTCCGCCCTGAGCAAAGACCCCAACGAGAAGCGCGATCACATGGTCCTGCTGGAGTTCGTGACCGCCGCCGGGATCACTCTCGGCATGGACGAGCTGTACATTCCTAAGAAGAAGAGAAAGGTTGAGGATTAAGCTTTGAGACC

PMC-01232

C-DsRed

GGTCTCAAGGTATGGGGTCATCCAAGAATGTTATCAAGGAGTTCATGAGGTTTAAGGTTCGCATGGAAGGAACGGTCAATGGGCACGAGTTTGAAATAGAAGGCGAAGGAGAGGGGAGGCCATACGAAGGCCACAATACCGTAAAGCTTAAGGTAACCAAGGGGGGACCTTTGCCATTTGCTTGGGATATTTTGTCACCACAATTTCAGTATGGAAGCAAGGTATATGTCAAGCACCCTGCCGACATACCAGACTATAAAAAGCTGTCATTTCCTGAAGGATTTAAATGGGAAAGGGTCATGAACTTTGAAGATGGTGGCGTCGTTACTGTAACCCAGGATTCCAGTTTGCAGGATGGCTGTTTCATCTACAAGGTCAAGTTCATTGGCGTGAACTTTCCTTCCGATGGACCTGTTATGCAAAAGAAAACAATGGGCTGGGAAGCCAGCACTGAGCGTTTGTATCCTCGTGATGGCGTGTTGAAAGGAGAGATTCATAAGGCTCTGAAGCTGAAAGACGGTGGTCATTACCTAGTTGAATTCAAAAGTATTTACATGGCAAAGAAGCCTGTGCAGCTACCAGGGTACTACTATGTTGACTCCAAACTGGATATAACAAGCCACAACGAAGATTATACAATCGTTGAGCAGTATGAAAGAACCGAGGGACGCCACCATCTGTTCCTTTAAGCTTTGAGACC

PMC-01302

C-mCherry

GGTCTCAAGGTATGGTGAGCAAGGGCGAGGAGGATAACATGGCCATCATCAAGGAGTTCATGCGCTTCAAGGTGCACATGGAGGGCTCCGTGAACGGCCACGAGTTCGAGATCGAGGGCGAGGGCGAGGGCCGCCCCTACGAGGGCACCCAGACCGCCAAGCTGAAGGTGACCAAGGGTGGCCCCCTGCCCTTCGCCTGGGACATCCTGTCCCCTCAGTTCATGTACGGCTCCAAGGCCTACGTGAAGCACCCCGCCGACATCCCCGACTACTTGAAGCTGTCCTTCCCCGAGGGCTTCAAGTGGGAGCGCGTGATGAACTTCGAGGACGGCGGCGTGGTGACCGTGACCCAGGACTCCTCCCTGCAGGACGGCGAGTTCATCTACAAGGTGAAGCTGCGCGGCACCAACTTCCCCTCCGACGGCCCCGTAATGCAGAAGAAAACCATGGGCTGGGAGGCCTCCTCCGAGCGGATGTACCCCGAGGACGGCGCCCTGAAGGGCGAGATCAAGCAGAGGCTGAAGCTGAAGGACGGCGGCCACTACGACGCTGAGGTCAAGACCACCTACAAGGCCAAGAAGCCCGTGCAGCTGCCCGGCGCCTACAACGTCAACATCAAGTTGGACATCACCTCCCACAACGAGGACTACACCATCGTGGAACAGTACGAACGCGCCGAGGGCCGCCACTCCACCGGCGGCATGGACGAGCTGTACAAGTAAGCTTTGAGACC

PMC-01118

T-35s

GGTCTCAGCTTCTCTAGCTAGAGTCGATCGACAAGCTCGAGTTTCTCCATAATAATGTGTGAGTAGTTCCCAGATAAGGGAATTAGGGTTCCTATAGGGTTTCGCTCATGTGTTGAGCATATAAGAAACCCTTAGTATGTATTTGTATTTGTAAAATACTTCTATCAATAAAATTTCTAATTCCTAAAACCAAAATCCAGTACTAAAATCCAGATCGCTTGAGACC

PMC-01319

T-OCS

GGTCTCAGCTTGTCCTGCTTTAATGAGATATGCGAGAAGCCTATGATCGCATGATATTTGCTTTCAATTCTGTTGTGCACGTTGTAAAAAACCTGAGCATGTGTAGCTCAGATCCTTACCGCCGGTTTCGGTTCATTCTAATGAATATATCACCCGTTACTATCGTATTTTTATGAATAATATTCTCCGTTCAATTTACTGATTGTACCCTACTACTTATATGTACAATATTAAAATGAAAACAATATATTGTGCTGAATAGGTTTATAGCGACATCTATGATAGAGCGCCACAATAACAAACAATTGCGTTTTATTATTACAAATCCAATTTTAAAAAAAGCGGCAGAACCGGTCAAACCTAAAAGACTGATTACATAAATCTTATTCAAATTTCAAAAGTGCCCCAGGGGCTAGTATCTACGACACACCGAGCGGCGAACTAATAACGCTCACTGAAGGGAACTCCGGTTCCCCGCCGGCGCGCATGGGTGAGATTCCTTGAAGTTGAGTATTGGCCGTCCGCTCTACCGAAAGTTACGGGCACCATTCAACCCGGTCCAGCACGGCGGCCGGGTAACCGACTTGCTGCCCCGAGAATTATGCAGCATTTTTTTGGTGTATGTGGGCCCCAAATGAAGTGCAGGTCAAACCTTGACAGTGACGACAAATCGTTGGGCGGGTCCAGGGCGAATTTTGCGACAACATGTCGAGGCTCAGCAGGACCGCTTGAGACC
